# The paradoxical role of urinary macromolecules in the aggregation of calcium oxalate: a further plea to increase diuresis in stone metaphylaxis

**DOI:** 10.1007/s00240-016-0863-z

**Published:** 2016-02-26

**Authors:** J. M. Baumann, B. Affolter

**Affiliations:** Stone Research Center Viollier, Biel, Switzerland

**Keywords:** Nephrolithiasis, Calcium oxalate aggregation, Albumin, Urinary macromolecules, Self-aggregation

## Abstract

This study was designed to get information on aggregation (AGN) of urinary calcium oxalate crystals (CaOx) which seems to occur in stone formation despite a protecting coat of urinary macromolecules (UMs). CaOx crystallization was directly produced in urine, control and albumin solution by Ox titration and was spectrophotometrically followed. A rapid decrease of optical density indicating AGN was absent in 14 of 15 freshly voided urines of 5 healthy controls. However, in the presence of UM-coated hydroxyapatite all urines with relative high sodium concentration, being an indicator of concentrated urine, showed a pronounced AGN which was abolished when these urines were diluted. Albumin relatively found to be an inhibitor of AGN showed after temporary adsorption on Ca Phosphate (CaP) massive self-AGN and changed to a promoter of CaOx AGN. Self-AGN after adsorption on surfaces especially of CaP, being an important compound of Randall’s plaques, can thus explain this paradoxical behavior of UMs. Aggregated UMs probably bridge zones of electrostatic repulsion between UM-coated crystals with identical electrical surface charge. These zones extend by urine dilution which decreases ionic strength. Diminution of urinary concentration by increasing diuresis seems, therefore, to be important in stone metaphylaxis.

## Introduction

Overgrowth of interstitial apatite plaques, so-called Randall’s plaques (RPs) or renal tubular crystal deposits by calcium oxalate (CaOx) seems to be important pathways in Ca nephrolithiasis [1–4]. RPs start their formation at the interior of the papillary tissue, initially without any contact with urine. Hydroxyapatite (HAP) calcification is induced by organic debris resulting from tissue injury. Later, when HAP deposits cross the epithelial monolayer that covers the papilla and enter in contact with urine, the apposition of CaOx crystals can occur. Large crystal aggregates being retained in collecting ducts and protruding out to papillary surfaces seem to be another starting point for stone formation. An initially fixed growth on such deposits or RPs allows stones to get a critical size where they cannot be washed out anymore from the kidney by the urine flow. Scanning electron microscopy of RPs mainly showed primary CaOx aggregates without direct contact to HAP particles and thus without an evidence for heterogenous nucleation of CaOx by HAP. Therefore, stone growth mainly seems to be based on crystal aggregation (AGN) on RPs, intratubular crystal plugs or preexisting stones during crystalluria [5, 6]. Since transit time of urine in the upper urinary tract is only in the order of a dozen of minutes, AGN has to occur very rapidly. Like in every biological fluid, urinary crystals are always coated by urinary macromolecules (UMs) [7]. UMs consist of a large group of proteins and some glycosaminoglycans [8]. The number of UMs isolated in urine is steadily increasing. From HAP precipitated in urine of healthy controls 45, and from brushite 77 different proteins were extracted [9]. The role of these proteins in stone formation is far from being clear. However, coating of crystals by UMs seems to prevent or at least retard AGN often beyond urinary transit time through the kidney [10]. This may explain why stone incidence and recurrence are less frequent than it could be expected from the widespread occurrence of RPs and crystalluria.

This paper tries to bring further light on crystal AGN in urine and the overgrowth of RPs or intra-tubular crystal deposits by CaOx, mechanisms still being poorly understood. To this purpose AGN of CaOx was measured in urine after rapid Ox titration in the presence and the absence of UM-coated HAP crystals. Since in previous work UMs showed some instability with a tendency to self-AGN [11], freshly voided spot urine was used having spent only a short time in the urinary tract. To mimic the influence of an increased diuresis, crystallization tests were repeated with diluted urine. Tests were also performed with UMs isolated from urine by Ca phosphate precipitation and consecutive dissolution of the precipitate. Furthermore, results were compared to experiments performed with albumin, being an important compound of crystal coats and stone matrix [8].

## Materials and methods

### Special equipment

Ionic Ca and Na concentrations were measured by ion-selective electrodes (AVL List GmbH). Light absorption or optical density (OD), respectively, in urine and solutions was measured at 620 nm and 37 °C in a Perkin Elmer spectrophotometer 550S (Perkin Elmer, Rotkreuz, Switzerland). OD was recorded with a DI-194RS serial port data recording module (DataQ Instruments, Ohio, USA) and for further calculation transferred to an Excel sheet. Particle size distribution was determined by a Malvern Zetasizer Nano ZS (Malvern Instruments Ltd).

### Preparation of urine, coated HAP crystals (cHAP), control (CS) and albumin solution (AS)

15 spot urines were collected every morning freshly from one of 5 healthy men. pH was adapted to 6.0, ionic sodium (Na^+^) measured and ionic calcium (Ca^2+^) adapted to 2 mM. In one portion of this urine a crystallization test was performed without further pretreatment. In a second portion 0.05 mg/mL hydroxyapatite (HAP) crystals (Sigma-Aldrich Co., Germany) was incubated under continuous stirring. The other urine samples were always diluted to 50 or 33 % with distilled water immediately before performing crystallization tests. After dilution pH was readapted to 6.0 and Ca^2+^ to 2 mM. CS was prepared in distilled water buffered with 5 mM sodium cacodylate to pH 6.0 and with concentrations of 100 mM Na^+^ and 2 mM Ca^2+^. HAP-saturated CS (HCS) was obtained by incubation of CS with 10 mg/mL HAP during at least 1 week and centrifugation at a relative centrifugal force (rcf) of 2000*g* for 10 min. AS was freshly prepared for every experiment dissolving powdered human serum albumin (Sigma-Aldrich Co., Germany) in CS or HCS to a final albumin concentration of 20 µg/mL. This concentration corresponds to a high physiological urinary excretion varying from 1.6 to 34 mg/day [8]. Albumin-coated HAP crystals were prepared in HCS as mentioned above for urine.

### Preparation of dissolved Ca phosphate precipitates (DP) from urine and albumin solution

2 mL of urine or AS was titrated in the spectrophotometer under continuous stirring at pH 7.0 by adding 0.5 mM/min of a 100 mM NaH_2_PO_4_ solution. The critical phosphate addition for an increase of OD was determined and calculated in mM. In 20 mL of new urine or AS, respectively, after adaptation of pH to 7.0 a phosphate load was performed, exceeding the critical addition for precipitation by 1.0 mM. After 30 min. of stirring, urine or AS was centrifuged and the supernatant discharged. The remaining sediment was dissolved in 20 mL of distilled water being buffered to pH 5.0. Complete dissolution was checked in the spectrophotometer. pH was adjusted to 6.0 and after measurement of Ca^2+^ and Na^+^ these values were adapted to 2 or 100 mM, respectively. Particle size distribution was measured in AS and in DP prepared from AS.

### CaOx crystallization test

CaOx crystallization was monitored by the spectrophotometer. Therefore, a quartz macro cuvette containing 2 mL of urine, CS or AS, respectively, was placed into the thermostattable cell holder of the spectrophotometer at 37 °C. Under continuous stirring 0.3 mM/min sodium oxalate was added from a 80 mM solution up to a final addition of 1.5 mM. At the end of the oxalate titration stirring was stopped and optical density (OD) was followed during further 25 min at 620 nm wavelength. To perform experiments with HAP, 2 mL of the 0.05 mg/mL HAP suspension in urine or HAP-saturated AS (HAS) was centrifuged (2000*g* for 10 min), the supernatant discharged and the sediment resuspended in 2 mL of new urine or HAS, respectively.

### Evaluation of crystallization curves

**Fig. 1:**
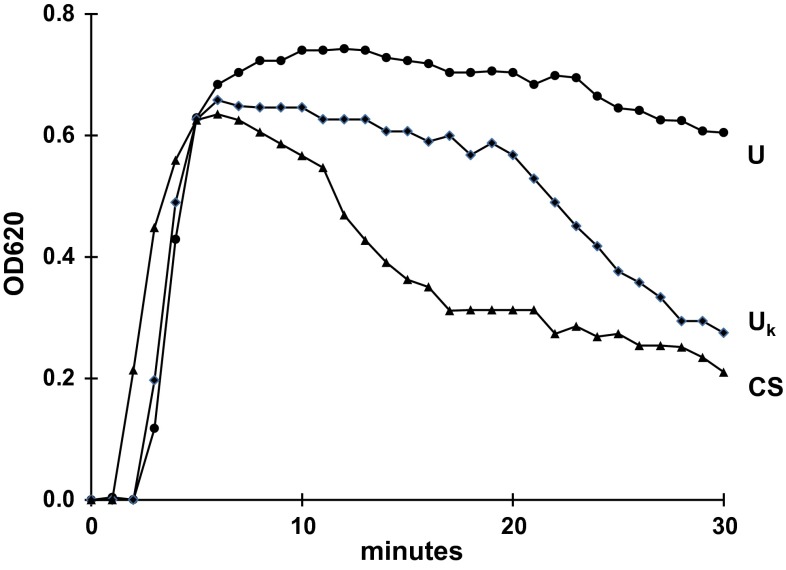
Crystallization curves represented by optical density (OD) in urine without (*U*), in urine with a kink in OD decrease (*U*_k_) and in control solution (CS), the kink indicating AGN

Characteristic crystallization curves are shown in Fig. 1. During Ox titration after a short period to reach the metastable limit, OD steadily increases to a maximal value (mOD) being proportional to the crystal concentration in the suspension [12]. At the end of Ox titration and after stopping stirring, two different patterns of OD decrease are observed. One type where in the sediment by scanning microscopy only single crystals are found shows a slow and continuous OD decrease. The other type where large crystal aggregates in the sediment are present, after a short period of slow OD decrease is characterized by a sharp kink with a rapid OD drop [13]. For the evaluation of crystallization curves, mOD and the maximal rate of OD decrease (mdOD/dt, min^−1^) were measured. OD decrease or the clearance of the particle suspension, respectively, occurs either when particles disappear by sedimentation out from the observation field of the spectrophotometer or when particle concentration or OD, respectively, is rapidly reduced by the association of many individual crystals in a few aggregates [10]. Results were indicated as mean ± SD and probabilities were calculated by Mann–Whitney *U* test.

## Results

### Evaluation of crystallization curves observed in undiluted urine

8 of 15 crystallization curves obtained in urine with coated HAP (cHAP) showed 10 ± 4 min after the stop of stirring a sharp kink of OD decrease indicating AGN. This kink as demonstrated in Fig. 1 also was found in CS. In 14 of 15 tests being performed without HAP only a slight and continuous OD decrease without a kink was observed.

Measurements of the crystallization parameters together with the initial urinary sodium concentration (Na^+^) are summarized in Table 1. Results are separately listed for urine samples without (*U*) and with a kink (*U*_k_) in the crystallization curve. The table shows that Na^+^ was significantly higher in *U*_k_ than in *U*. The maximal OD reached after Ox titration (mOD) reflecting crystal concentration was generally higher in urine than in CS. The difference mainly can be attributed to the additional Ox brought with urine to the test system. A significantly elevated maximal rate of OD decrease (mdOD/dt) indicating AGN was, as could be expected from the above-mentioned observation, exclusively found in experiments performed in *U*_*k*_ containing cHAP. Values were in the range of those found in CS, where the addition of HAP produced only a slight and non-significant further increase of mdOD/dt. Contrary to results obtained in CS almost all crystallization tests performed in urine without cHAP showed a low mdOD/dt indicating inhibition of AGN. cHAP at high Na^+^, the latter being an indicator for concentrated urine [14], seems to overwhelm this inhibition and promote CaOx AGN. For further investigation crystallization experiments were repeated after dilution of *U*_k_ samples.

**Table 1:** Urinary sodium concentration (Na, mM), maximal optical density (mOD) and maximal rate of OD decrease (mdOD/dt, min^−1^) in *U*, *U*_k_ and CS (for further details see Fig. 1)

	*U*	*U*_k_	CS	*p*
Na^+^	67.9 ± 22.6^a)^	125.3 ± 17.2^b)^	100	(a) vs (b) <0.01
mOD	0.77 ± 0.08^a)^	0.86 ± 0.08^b)^	0.55 ± 0.09^c)^	(a) vs (c) <0.01 (b) vs (c) < 0.01
mOD+	0.73 ± 0.08^a)^	0.77 ± 0.18^b)^	0.43 ± 0.05^c)^	(a) vs (c) <0.01 (b) vs (c) <0.05
mdOD/dt	0.008 ± 0.002^a)^	0.011 ± 0.004^b)^	0.033 ± 0.008^c)^	(a) vs (c) <0.01 (b) vs (c) <0.01
mdOD/dt+	0.011 ± 0.004^a)^	0.034 ± 0.011^b)^	0.044 ± 0.013^c)^	(a) vs (b) <0.01 (a) vs (c) <0.01

Experiments performed in the presence of coated HAP are indicated by (+). Results (mean ± SD) are marked by a, b and c to indicate probability (p) of differences

### Effect of dilution

**Fig. 2:**
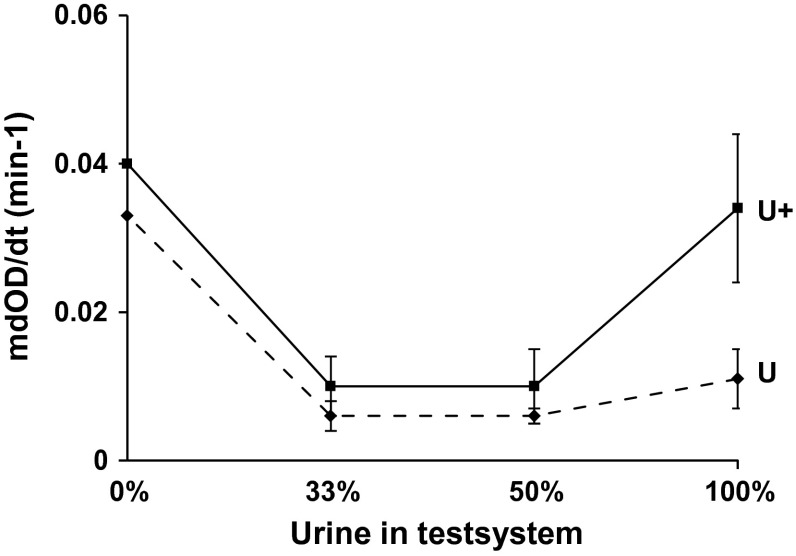
Maximal rate of OD decrease (mdOD/dt, min-1) of *U*_*k*_ with (*U*+) and without coated HAP (*U*) at different states of dilution indicated as percent of urine in the test system

Figure 2 shows the effect of the dilution of initially concentrated *U*_k_ on mdOD/dt. The figure demonstrates that dilution significantly (*p* < 0.01) reduced the elevated mdOD/dt observed in the concentrated *U*_k_ containing cHAP. The inhibition of AGN observed in tests performed without cHAP was not significantly changed even by an urine dilution down to 33 %. These experiments confirmed that a high urine concentration together with cHAP was responsible for CaOx AGN and furthermore showed that this cHAP-induced AGN could be prevented by urine dilution. Therefore, the question rises whether at high urinary concentration a weak promoter becomes active or inhibitory substances turn to promoters.

### Comparison of tests performed with *U*_k_ and albumin solution (AS) and with dissolved Ca phosphate precipitates (DP) from *U*_k_ and AS

**Fig. 3:**
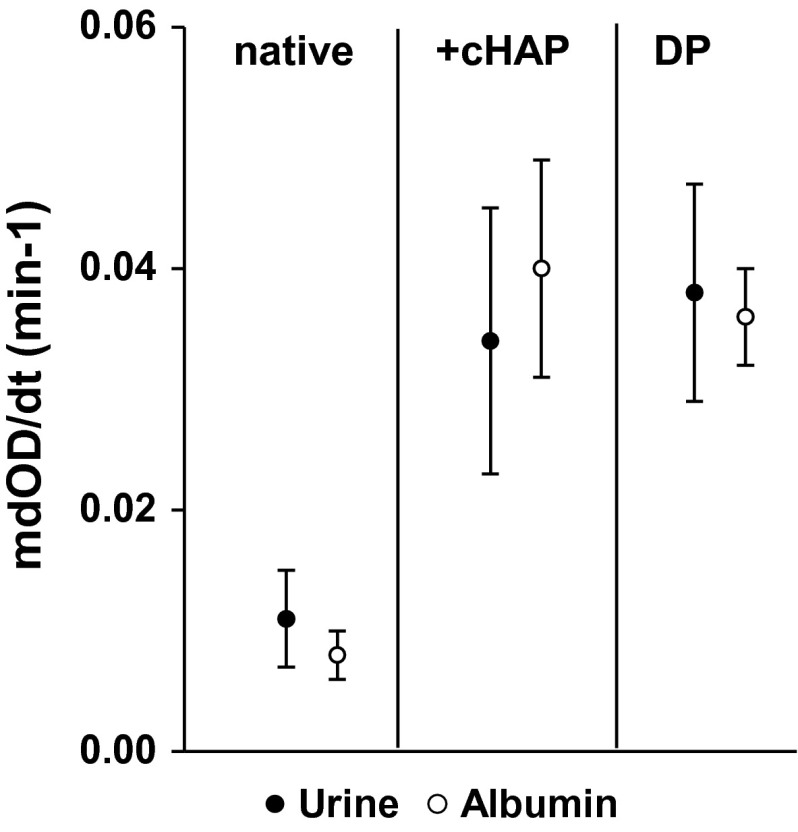
Comparison of mdOD/dt observed in *U*_k_ and albumin solution (AS, 20 µg/mL) without (native) and with coated HAP (+cHAP) and in the dissolved Ca phosphate precipitate (DP) from *U*_*k*_ and AS

**Fig. 4:**
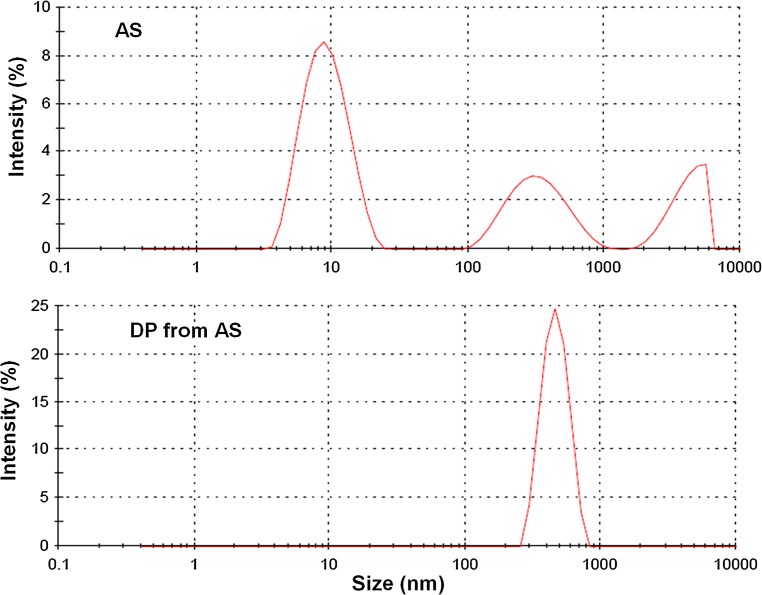
Particle size distribution of AS and of DP from AS

*U*_k_ and AS in high physiological concentration of 20 µg/mL showed, as Fig. 3 demonstrates, an almost identical behavior with respect to mdOD/dt. Both revealed without cHAP a very low mdOD/dt indicating an excellent inhibition of AGN. Exposition of *U*_k_ and AS to pre-incubated HAP and the extract from *U*_k_ and AS in DP produced the same pronounced increase of mdOD/dt (*p* < 0.01). Albumin being the only crystallization modulator in the corresponding experiments changed, thus by adsorption on Ca phosphate, from an inhibitor to a promoter of CaOx AGN. Analysis of particle size distribution of AS showed apart from the main peak at 10 nm further smaller peaks of higher particle size, demonstrating some self-AGN. In DP, after temporary adsorption on Ca Phosphate, all albumin self-aggregated to a small single peak with a maximum at 470 ± 14 nm (Fig. 4).

## Discussion

This paper tries to give some answers to how urinary CaOx crystals especially in the presence of HAP can aggregate despite of their UM coat and how this AGN could be prevented. To induce spectrophotometrically measurable AGN within a time being similar to the generally short urinary transit time through the kidney, spot urine of healthy controls was rapidly titrated by a relative high dose of 1.5 mM Ox. In a previous study performed after thawing of frozen urine, this Ox dose was able to induce CaOx AGN in 10 urine samples of 30 healthy controls and in 20 of 30 stone patients [10]. Interestingly, in the present study under almost identical conditions only in 1 of 15 freshly voided urine samples AGN was observed. These urines revealed thus a high inhibitory activity with respect to CaOx AGN which after freezing and thawing partially was lost. However, HAP crystals which previously were incubated in urine induced CaOx AGN in all urine samples with a relative high Na^+^. A high Na^+^ as mentioned above is an indicator for concentrated urine. Dilution of urine abolished HAP-induced AGN but did not diminish the inhibitory activity with respect to pure CaOx crystallization even at urinary concentration in the test system of only 33 %. This agrees with the findings of others that urine of healthy subjects diluted to 20 % strongly inhibited CaOx AGN [15]. Furthermore, a study of CaOx crystallization in urine of stone patients and controls under different states of diuresis revealed an inverse relationship between urine volume and the intensity of AGN [14]. The paradoxical fact that the dilution of urinary inhibitors prevented AGN was addressed but could not be explained by this study.

AGN generally is ascribed to the attraction of particles by Van der Waal’s forces (VWF) which are only effective at very short distances [16]. Other factors are viscous binding and solid bound formation. The first is a rapid process, the latter is slower as it requires deposition of further crystal material between already formed crystals. Particles like CaOx crystals have an electronegative surface charge which by electrostatic repulsion of the identically charged crystals normally prevents their AGN. By Ca addition to CaOx suspensions this surface charge was neutralized [17] and at sufficient crystal concentration, as demonstrated by our experiments performed in CS, crystals rapidly aggregated. In urine crystals normally are protected by a UM coat with a thickness of 10–20 nm [7] and with an electronegative potential in the order of −15 mV [13]. Such potentials are able to counteract VWF by repulsion of the identically charged particles [16]. However, as mentioned above, after high oxalate additions or at high crystal concentrations, respectively, CaOx AGN also occurred in urine.

Three different theories try to explain the AGN of UM-coated particles: incomplete coating of crystals, insufficient surface potential of coats and bridging between crystals by altered proteins being called viscous binding [5]. Scanning microscopy of crystal aggregates being produced in protein solutions showed gaps in protein coats where some crystals were aggregated in direct contact with each other [7]. But it could not be decided whether crystal coating had occurred before or after AGN and in other aggregates at points of crystal convergence large amorphous material was observed suggesting a bridging function of protein. The electronegative charge of UMs can be attributed to anionic residues like carboxyglutamic acid [18, 19], phosphate [20–22] and sialic acid [23, 24] which were found reduced in UMs of some stone patients. A lack of electrostatic repulsion is, therefore, often claimed to be responsible for AGN of urinary crystals. However, a reduction of anionic groups also enhances the hydrophobic effect in UMs which can provoke self-AGN. This was demonstrated by desialylation of Tamm Horsfall protein (THP) [24], an important UM involved in crystal adherence to RPs [2]. Normal THP in high concentration and at low pH, high ionic strength and high Ca concentration too tend to self-AGN and promote CaOx AGN [25, 26]. This promotion probably bases on a bridging function. In electrolyte containing solutions, surface potentials rapidly decrease with increasing distance from negatively charged particles by cation accumulation in their surroundings [16]. In urine with increasing concentration and ionic strength surface potentials are compressed to a few nanometers. Identically charged particles can, therefore, approach each other to a critical distance where diffusion, sedimentation or mechanic forces like stirring or shaking are compensated by the electrostatic repulsion. Large UM aggregates probably are able to bridge such zones of repulsion and to bind to crystal coats by hydrophobic effects [5].

The almost identical behavior of concentrated urine and albumin solution (AS) in our crystallization experiments showed that albumin is an ideal compound to mimic the overall effect of UMs under our special test conditions. In the presence of coated HAP (cHAP) the inhibition of CaOx AGN turned in both mediums to promotion. This effect was not directly related to cHAP since it was also observed in the dissolved Ca Phosphate precipitates (DP) of urine and AS. In this respect it is interesting to note that the formation of HAP in some loops of Henle with an urinary pH of 7.4 appears to be a normal phenomenon and that this HAP then dissolves in the distal part of the nephron where pH decreases [27]. Under these conditions the promoting effect of temporarily adsorbed UMs may persist and may favor the AGN of newely formed crystals in the distal nephron. A promoting effect on crystal AGN was also found in UMs isolated from urine by a hemofiltration procedure [11]. Crystal deposits as well as hemofilters provide a large surface for adsorption. Ca phosphate as demonstrated by the adsorption of 77 different proteins on brushite and of 45 on HAP [9] has a special ability for protein accumulation which may explain the essential role of HAP containing Randall’s plaques (RPs) in idiopathic Ca nephrolithiasis [2]. With respect to albumin it could be demonstrated that adsorption on Ca phosphate produced albumin aggregates with an average diameter of 470 nm largely being able to take over a bridging function. However, in our study such a bridging probably was counteracted by urine dilution which by diminishing ionic strength increases the radius of electrostatic repulsion and thus the distance between crystals to be bridged for AGN. On the other hand, our experiments performed with cHAP suggests that at a critical urinary concentration RPs and intratubular crystal deposits being coated by aggregated UMs are ideal platforms for stone growth by crystal AGN during crystalluria. Scanning electron microscopy of urinary sediments performed after HAP-induced CaOx AGN showed in agreement with findings on RPs large CaOx aggregates which were in the surroundings of HAP but not in direct contact with HAP crystals [28]. HAP thus seems not to act as nucleator of CaOx crystallization in urine but as mediator for the self-AGN of UMs which promote crystal AGN.

## Conclusions

Our findings give further evidence that CaOx AGN in urine probably is mediated by self-aggregated UMs forming bridges between UM-coated crystals. Whether pathological UMs or as our study suggests a pathological urine concentration is more relevant for CaOx AGN cannot be decided. However, the observation that crystal AGN is enhanced by a high urine concentration is a further argument in stone metaphylaxis to increase diuresis as being already established to diminish urinary supersaturation as well as urinary transit time in the renal collecting system. The influence of freezing and hemofiltration on results of crystallization experiments demonstrates that tests performed in freshly voided and unpretreated urine are important for the study of inhibitors and promoters in stone research.
